# MicroRNA-21 as a diagnostic marker for hepatocellular carcinoma: A systematic review and meta-analysis

**DOI:** 10.12669/pjms.35.5.685

**Published:** 2019

**Authors:** Juan Qu, Jizhi Yang, Ming Chen, Lihong Cui, Tianxi Wang, Wei Gao, Jingjing Tian, Rongna Wei

**Affiliations:** 1 Juan Qu, Department of Gastroenterology, Tianjin Nankai Hospital, Tianjin 300100, China; 2 Jizhi Yang, Department of Traditional Chinese Medicine, Chentangzhuang Hospital, Hexi District, Tianjin 300222, China; 3 Ming Chen, Department of Hepatopathy and Hepatic Oncology, Tianjin Nankai Hospital, Tianjin 300100, China; 4 Lihong Cui, Department of Gastroenterology, Tianjin Nankai Hospital, Tianjin 300100, China; 5 Tianxi Wang, Department of Gastroenterology, Tianjin Nankai Hospital, Tianjin 300100, China; 6 Wei Gao, Department of Gastroenterology, Tianjin Nankai Hospital, Tianjin 300100, China; 7 Jingjing Tian, Department of Gastroenterology, Tianjin Nankai Hospital, Tianjin 300100, China; 8 Rongna Wei, Department of Gastroenterology, Tianjin Nankai Hospital, Tianjin 300100, China

**Keywords:** Biomarker, Early diagnosis, Hepatocellular carcinoma, microRNAs

## Abstract

**Background:**

MicroRNA-21 (miR-21) is one of the oncogenic miRNAs which may be a potential diagnostic biomarker for hepatocellular carcinoma (HCC).

**Methods:**

We systematically searched Medline, Embase, the Cochrane Library, ISI Web of Knowledge, Scopus from inception to August 15, 2018, and reference lists of identified primary studies. Two independent investigators extracted patient and study characteristics. The sensitivity and specificity of microRNA-21 for HCC detection and were analyzed with a random effect model. The area under summary receiver operating characteristic curve (AUC) was used to estimate overall test performance.

**Results:**

A total of 515 HCC patients, and 338 healthy or chronic hepatitis controls from six published studies were enrolled in this meta-analysis. All articles were published in English with moderate-to-high quality. The overall pooled sensitivity and specificity were 85.2% (73.3% to 88.4%) and 79.2% (68.4% to 87.0%), respectively. The AUC area was 0.89 (95% CI: 0.85-0.91). The studies had moderate heterogeneity (I2=70.11%). None of the subgroups investigated—ethnicity, controls, sample source—could account for the heterogeneity.

**Conclusion:**

MiR-21 is a helpful biomarker for early diagnosis of HCC. Nevertheless, the results of the test must be interpreted carefully in the context of medical history, erological tests and imaging examinations for HCC surveillance.

## INTRODUCTION

Primary liver cancer is one of the most common malignant tumors and the second leading cause of cancer-related mortality worldwide.^1^ The incidence of liver cancer continues to increase rapidly and the death rates rose for liver cancer by 2.7% per year in women and by 1.6% per year in men during 2011 through 2015.^2^ Hepatocellular carcinoma is the most common histologic type of primary liver cancer, accounting for more than 90%. Patients with HCC with early diagnosis have good prognosis after a curative operation, and 5-year overall survival rate can reach 50–74%.^3^ However, the one-year survival rate is lower than 10% for patients with widespread cancer, and the overall 5-year survival for HCC is still less than 10% globally.^3^ Therefore, early diagnosis is of great importance to improve survival of HCC.

Currently, early detection of HCC mainly relies on erological tests and imaging examinations. Ultrasonography (US) is the most commonly used imaging examinations with relative low cost, but has low sensitivity (63%) in early detection of HCC.^4^ Serolgocial test for α-fetoprotein (AFP), AFP-L3, des-gamma-carboxy prothrombin (DCP) have been investigated for HCC diagnosis alone or in combine. However, these tests have been shown to be suboptimum for routine monitor of HCC, and the most widely used tumor marker AFP is not secreted in all hepatocellular carcinomas.^5^ Though serum AFP at a cutoff of 20 ng/mL has a sensitivity of 40–65% for clinically diagnosed HCC, only 14–40% of them are with preclinical disease.^6^ Similar to AFP-L3, the specificity and sensitivity of DCP for HCC diagnosis ranges from 36% to 96% and 89% to 94%.^6^

MicroRNAs (miRNAs) are small non-coding RNAs which were involved in human carcinogenesis by regulating specific target genes. Aberrant expressions of miRNAs have been reported to play important roles the development of various cancers.^7^ It was supposed that miRNAs could be stably detectable in plasma/serum. Besides, serum and plasma samples are relatively easy to acquire. Thus, circulating miRNAs can serve as potential biomarkers for cancer diagnosis.^8^-^10^ MiR-21 is one of the oncogenic miRNAs widely studied in a number of cancers.^11^-^14^ It is involved in cell proliferation, migration and apoptosis, and could promote invasion and metastasis in human cancers.^15^ The diagnostic role of circulating miR- 21 has been widely studied in various human malignant cancers.^16^-^19^ In the publication of Liao’s^20^ meta-analysis, they investigated the sensitivity and specificity of miR-21 as a biomarker in the diagnosis of HCC. However, relative researches are really limited and only four articles were included (one in Chinese). Considering the limits of existing publications, we conducted a novel meta-analysis of miR-21 for HCC including newly published researches to obtain a better understanding of the diagnostic efficiency of miR-21 in HCC.

## METHODS

### Search strategy

We systematically searched the following databases including: Medline (via PubMed), Embase (via Ovid), the Cochrane Library, ISI Web of Knowledge and Scopus for articles published up to August 15, 2018.

In search of studies that assessed the accuracy of miR-21 for the diagnosis of HCC, the terms for literature retrieval were used as follows: (“liver neoplasms” or “liver neoplasm” or “hepatic neoplasm” or “liver cancer” or “hepatocellular cancer” or “hepatic cancer” or “cancer of the liver” or “hepatocellular carcinoma”) and (microRNA-21 or miRNA-21 or miR-21 or has-miR-21). When searching ISI Web of Knowledge and Scopus, we also used the search terms “NOT (letter OR review OR editorial OR “animal experiment” OR “meeting abstract” OR “proceeding paper” OR “poster presentation” OR “meta-analysis” OR “case report”)” to reduce the number of unrelated results. To identify additional relevant studies, we also examined the reference list of previous systematic reviews and primary studies.

### Selection criteria

Studies were included if they met the following inclusion criteria:

1. Definitive diagnosis of HCC using gold standard.
2. miR-21 expression in plasma, serum, feces or tissues was detected.
3. Sufficient data for constructing the 2×2 contingency table, i.e., true positive (TP), false positive (FP), false negative (FN), and true negative (TN) were provided.

Besides, we only included publications written in English. Letters, animal experiment, reviews, cases reports, conference abstract, expert opinions and editorials were excluded. We also excluded studies with unqualified data.

### Data extraction and quality assessment

Two investigators (Juan Qu and Jizhi Yang) were responsible for data extraction independently. The following data were extracted: first author, publication year, country, ethnicity of participants, number of participants, source of samples, and diagnostic results including details of the miR-21 assays and cutoffs used, sensitivity, specificity, true positives (TP), false negatives (FN), false positives (FP), and true negatives (TN). Any discrepancy between the two investigators was solved by a consensus meeting or referral to a third investigator (Ming Chen). The quality assessment of the selected studies was performed using the Quality Assessment of Diagnostic Accuracy Studies (QUADAS) checklist.^21^,^22^

### Statistical analysis

The number of patients with true positives (TP), false negatives (FN), false positives (FP), and true negatives (TN) from the enrolled studies were extracted for the diagnostic meta-analysis. The bivariate meta-analysis model was applied to generate the bivariate summary receiver operator characteristic (SROC) curve and calculate the pooled parameters including the sensitivity, specificity, positive likelihood ratio (LRP), negative likelihood ratio (LRN), diagnostic odds ratio (DOR).^23^,^24^ We assessed heterogeneity using the I-squared index and χ2 test and heterogeneity existed when I2 > 50% and/or p < 0.05.^25^ To evaluate the potential publication bias, we used Deeks’ funnel plot asymmetry test, and a p value <0.1 was considered to indicate that as significant publication bias existed among the enrolled studies. We further performed meta-regression to explore the source of heterogeneity. All the statistical analyses were carried out using Stata 12.0 and Meta-DiSc 1.4.^26^

## RESULTS

### Study selection and quality assessment

**Fig.1:**
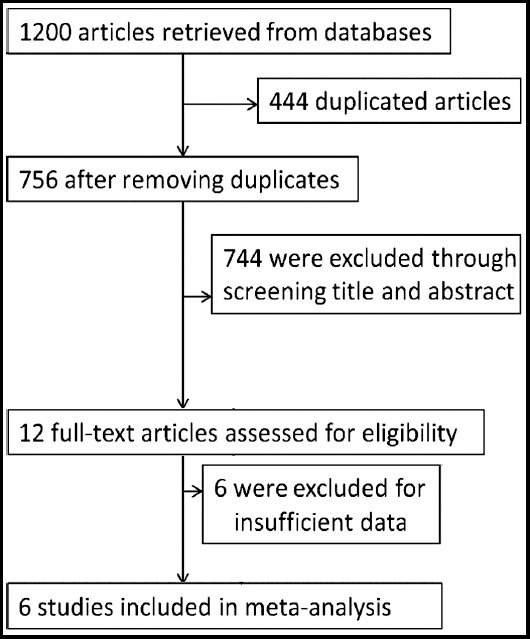
Flow diagram of the study selection process.

The literature search retrieved 1200 relevant articles, and 444 duplicate publications were excluded. After preliminary reviewing the titles and abstracts, we excluded 744 articles because they were reviews, cases, letters, conference abstract, or studies not relevant. After a full text review, we excluded 6 articles for insufficient data, leaving six studies for inclusion (Fig.1). One relevant study was identified through searching the reference list of the previous systematic reviews and related articles.^27^-^32^

The main characteristics of the selected publications are shown Table-I. A total of 823 subjects were included in the analysis, of which 515 had HCC, and 308 were healthy or chronic hepatitis controls. All articles were published in English. The gold standard for HCC diagnosis was histopathological examination. The level of miR-21 was detected by real-time quantitative PCR (RT-PCR). The quality assessment of the selected studies using QUADAS criteria is shown in Supplementary Fig.1, suggesting a moderate-to-high quality of the selected studies.

**Table I:** Main characteristics of the studies included in the meta-analysis.

Author	Year	Country	Ethnicity	Case (n)	Control	No. of control (n)	Sample	AUC	Sensitivity (%)	Specificity (%)
Amr	2016	Egypt	Caucasian	23	Patients with chronic hepatitis	17	Serum	0.943	100	81.2
Gedawy	2017	Egypt	Caucasian	30	Chronic liver diseases	20	Plasma	NA	93	90
Tomimaru	2012	Japan	Asian	126	30 chronic hepatitis (CH)	30	Plasma	0.773	61.1	83.3
Tomimaru	2012	Japan	Asian	126	50 healthy volunteers (HVs)	50	Plasma	0.953	87.3	92
Xu	2011	China	Asian	101	89 Healthy controls	89	Serum	0.87	84	73.5
Zhuang	2016	China	Asian	52	43 healthy controls	43	Serum	0.621	67.4	55.8
Liu	2012	China	Asian	57	59 hepatitis B carrier/healthy controls	59	Serum	0.865	89.47	71.19

### Diagnostic accuracy of miR-21 in HC

**Fig.2:**
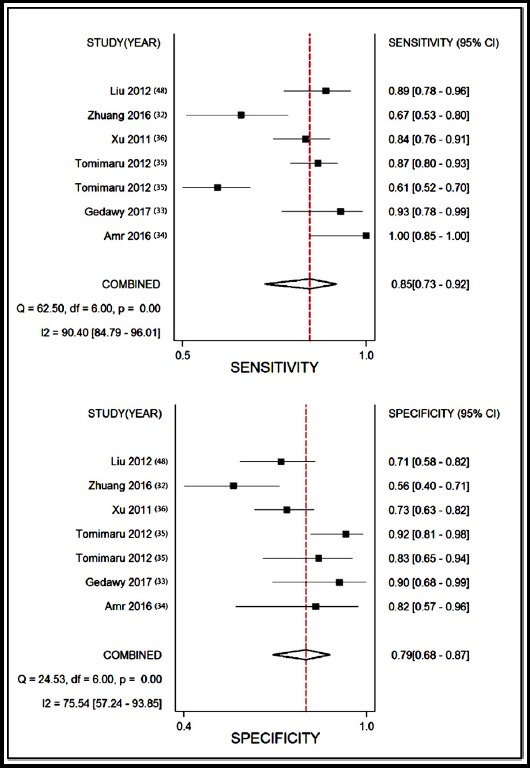
Sensitivity and specificity of circulating miR-21 for diagnosis of HCC.

**Fig.3:**
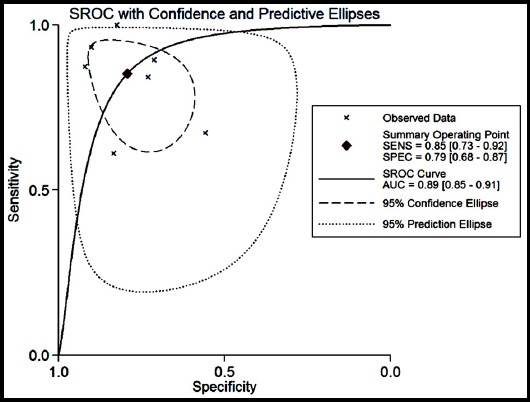
Summary receiver operating characteristic curve

Moderate heterogeneity was observed with χ2 = 6.691, p = 0.018; I^2^ = 70.11%. Thus, we selected the random effects model. The pooled sensitivity and specificity of the enrolled studies are summarized in Fig.2. Moderate to significant heterogeneity exists among for I^2^ values in sensitivity (90.4%) and specificity (75.54%). The overall pooled sensitivity and specificity were 85.2% (73.3% to 88.4%) and 79.2% (68.4% to 87.0%), respectively. The diagnostic odds ratio (DOR) was 21.970 (95% CI: 7.433-64.944), which suggesting a chance of a 21.970-fold higher level of miR-21 in subjects with positive HCC diagnosis compared with subjects with negative results. The AUC area was 0.89 (95% CI: 0.85-0.91) as shown in the summary receiver operator characteristic (SROC) curve (Fig.3), revealing overall moderate diagnostic accuracy. The combined positive likelihood ratio (PLR) and negative likelihood ratio (NLR) were 4.098 (95% CI: 2.493-6.735) and 0.187 (95% CI: 0.094-0.369), respectively.

### Threshold effect

The differences in sensitivities and specificities are the source of threshold effect. In the present study, the threshold effect was assessed with Spearman correlation coefficient. A value of -0.214 (p=0.645; p>0.05) suggested that no evidence of threshold effect existed in this meta-analysis.

### Subgroup and meta-regression analysis

Metaregression analyses were then carried out to identify the source of heterogeneity. We found that the pooled sensitivity and specificity of the studies were 0.962 (95% CI: 0.870-0.995) and 0.865 (0.712-0.955) for Caucasian populations *versus* 0.775 (95% CI: 0.734-0.812), and 0.745 (0.689-0.796) for Asian populations, with no significant difference (p>0.05). Subgroup analysis by sample type (serum or plasma) found that no significant difference was observed in the diagnostic accuracy between miRNA-21 levels in serum and plasma, with sensitivity of 0.833 (95% CI: 0.778-0.878) versus 0.762 (0.708-0.811), specificity of 0.697 (0.630-0.759) versus 0.890 (0.812-0.944). The summary sensitivity and specificity of circulating miR-21 for discriminating HCC from healthy individuals were 83.6% (79.2-87.4) and 73.4% (67.4-78.9), respectively. In contrast, the pooled sensitivity and specificity of circulating miR-21 for discriminating HCC from chronic hepatitis were 75.8% (69.9-81.2) and 78.6% (70.4-85.4), respectively.

### Publication bias

**Fig.4:**
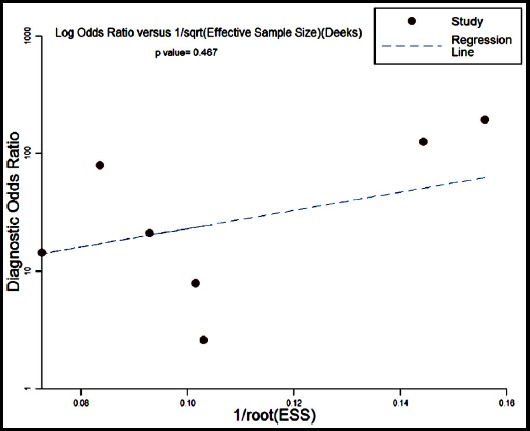
Deeks’ funnel plots for the assessment of potential publication bias.

The potential publication bias was explored using Deeks’ funnel plots in this meta-analysis. The obtained p-value of 0.467 indicated that there was no publication bias (Fig.4).

## DISCUSSION

Tumor biomarkers are critical in cancer diagnosis, especially those noninvasive ones.^33^ MiRNAs are regarded as perfect diagnostic markers for cancers as they are stably detectable in plasma/serum which relatively easy to acquire.^34^ Besides, miRNAs are involved in a variety of important biological processes, such as cell proliferation, migration, and apoptosis^35^ MiR-21 is one of the widely-studied miRNAs which is potential biomarker for HCC.^32^,^36^ However, some inconsistent findings were generated from a series of quantitative analyses, proposing the necessity of conducting meta-analysis and systematic review to investigate the diagnostic value of miR-21 in HCC.

The present meta-analysis showed that miR-21 presented diagnostic sensitivity of 85.2%, (73.3% to 88.4%), which was superior in HCC diagnosis as compared with AFP whose overall diagnostic sensitivity was less than 60%.^37^ In addition, the sensitivity was also higher than computed tomography (CT) or magnetic resonance imaging (MRI). The AUC of miR-21 was 0.89 (95% CI: 0.85-0.91), which was also slightly higher than that of AFP (0.81). These two representative parameters in combination with the specificity of 79.2% indicated an overall moderate diagnostic value of miR-21 as a promising noninvasive marker for HCC diagnosis. As a potential diagnostic biomarker for HCC, miR-21 has many unique advantages as compared with histopathological examination or AFP: (1) minimal invasiveness and convenience with no need of invasive or harmful procedures to obtained sample, (2) stability and reproducibility^8^ and early expression in HCC patients.^31^ In addition, as a marker for HCC, AFP level of 400 ng/ml is regarded as a threshold for screening of HCC patients. However, in about one-third of all HCC case with small lesions (<3cm), AFP level does not reach such value at an early HCC stage, leading to missed diagnosis in their early tumor stage.^38^ Nevertheless, considering the thresholds of PLR > 10 and NLR < 0.1 indicating high accuracy, the values for PLR (4.098) and NLR (0.187) in the present meta-analysis suggested caution regarding the diagnostic power of miR-21 for HCC screening alone. Taken together, circulating miR-21 may be a novel cobiomarker which may increase the diagnostic accuracy of early-stage HCC.

Moderate heterogeneity was discovered in this meta-analysis (I^2^ = 70.11%). No heterogeneity was caused by the threshold effect, indicated by the Spearman’s correlation coefficient of 0.645 (p > 0.05). Meanwhile, after assessing the effects of ethnicity, sample source and type of controls in diagnostic accuracy, meta-regression failed to identify potential sources generating the heterogeneity among the included studies. An absence of publication bias was also revealed by the funnel plot.

### Limitations in the present study

First, over a half of the included studies use healthy controls and this limits the diagnostic performance. Second, the over-expression of circulating miR-21 is not uniquely detected in HCC. It was also reported in other human tumors, such as colorectal, digestive and lung cancers.^19^,^39^,^40^ Thus, miR-21 must be used combined with other marker of HCC diagnosis in routine clinical practice. As reported by Tomimaru et al., the combination of plasma miR-21 and AFP has an AUC of 0.971 in discriminating HCC from healthy controls, and an AUC of 0.823 in discriminating HCC from patients with chronic hepatitis^31^, which has far better performance than AFP alone. It has been well-known that high incidence of HCC is observed in Asian countries, such as China and Japan.^1^ As a consequence, most studies included in this meta-analysis were originated from China and Japan. Limited studies based on Caucasians and no African populations were enrolled.

## CONCLUSIONS

This meta-analysis assessed the application of circulating miR-21 for HCC diagnosis. Our results reveal that circulating miR-21 has an overall moderate diagnostic performance, and can be used as a potential noninvasive marker for early-stage HCC diagnosis. Further large-scale prospective studies are needed in order to validate the clinical application of miR-21 and develop better diagnostic models with more prediction capacity.

### Author’s Contribution:

**QJ** conceived and designed the study and drafted the manuscript; **QJ and YJ** were responsible for data extraction. **CM** was responsible for checking data, and resolving discrepancies during data extraction. **YJ, CM and CL** were responsible for data analysis. **WT and GW** were responsible for literature retrieval and study selection. **TJ and WR** were responsible for study selection. **QJ** takes the responsibility and is accountable for all aspects of the work in ensuring that questions related to the accuracy or integrity of any part of the work are appropriately investigated and resolved.
